# Anaesthesia and environment: impact of a green anaesthesia on economics

**DOI:** 10.1097/ACO.0000000000001243

**Published:** 2023-01-23

**Authors:** Jasper M. Kampman, Nicolaas H. Sperna Weiland

**Affiliations:** a Amsterdam UMC location University of Amsterdam, Anaesthesiology; b Amsterdam UMC Centre for Sustainable Healthcare, Amsterdam, The Netherlands

**Keywords:** climate change, economics, environmental sustainability, waste reduction

## Abstract

The excessive growth of the health sector has created an industry that, while promoting health, is now itself responsible for a significant part of global environmental pollution. The health crisis caused by climate change urges us to transform healthcare into a sustainable industry. This review aims to raise awareness about this issue and to provide practical and evidence-based recommendations for anaesthesiologists.

**Recent findings:**

We offer seven recommendations for anaesthesiologists that want to transform their own practice.

1. Use total intravenous anaesthesia over inhalation-based anaesthesia.
2. Use ultra-low fresh gas flow (<0.5 l min^−1^) during inhalation based anaesthesia and higher flows (4–6 l min^−1^) during total intravenous anaesthesia.
3. Anaesthetic gas capturing technology may have limited environmental benefit, while it increases cost.
4. In the clinical setting, epidural or intravenous analgesia during labour is preferred over nitrous oxide inhalation.
5. Properly designed air treatment systems save energy and reduce cost.
6. Unoccupied set-back of air treatment systems can save up to 70% of energy.
7. Reusable equipment is almost always associated with a lower environmental impact and lower costs compared with single-use disposables.

**Summary:**

This review offers evidence-based recommendations, along with their financial impact, to improve the sustainability of anaesthesiology practice in the operating room.

## INTRODUCTION

**FIGURE 1:**
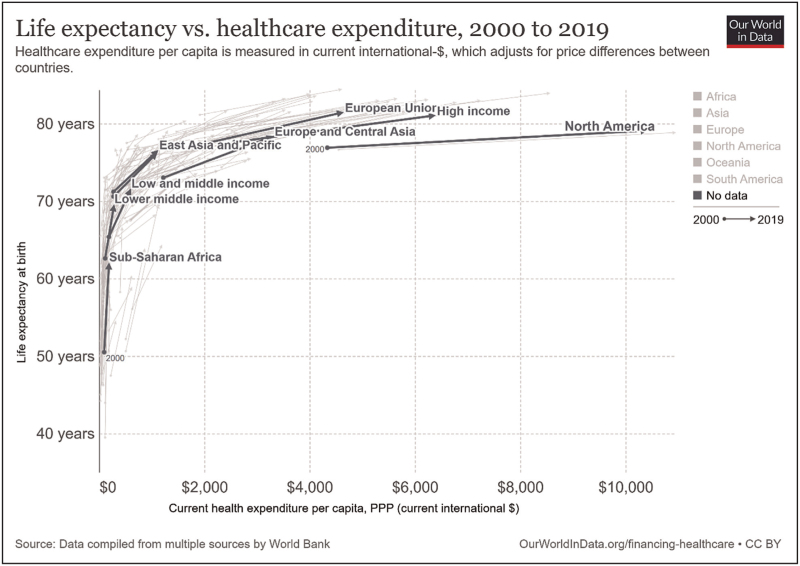
Relation between life expectancy and healthcare expenditure between 2000 and 2019. For the higher-income regions it shows clear signs of ‘the law of diminishing returns’. This law describes the situation where initial input (i.e. the left side of the curve) leads to significant improvements in output (e.g. patient outcomes), but where the effectiveness of additional input (e.g. health expenditure, or tests/interventions) diminishes when the curve approaches the ‘flat of the curve’. In theory, additional input will at some point lead to patient harm. Figure source: Our World In Data [3].

Global life expectancy at birth has more than doubled from about 29 years in 1800, to 67 years in 2001 [1]. This phenomenon is strongly associated with growing economic power [2], but also with technological progress and increased scientific understanding [3]. Modern medicine has made important contributions through propagation of hygiene measures, the development of vaccinations and therapies against infectious diseases and cardiovascular disease [4]. Often overlooked in this matter is that surgical care has greatly reduced maternal and child mortality and that improving access to surgical care in lower- and middle-income countries may be one of the most cost-effective public health interventions today [5,6]. Conversely increased spending on healthcare in high-income countries clearly suffers from ‘the law of diminishing returns’ and these countries are struggling to justify allocating increasing parts of their annual budget to healthcare (Fig. 1) [7,8].

As a basic law in economics, increased expenditure translates into increased demand for natural resources and labour capacity. Also, economic ‘externalities’ such as environmental pollution with greenhouse gases (GHGs) and waste generation have long been neglected. It is therefore not surprising that a large industry as the healthcare sector is responsible for 4.4% of GHG emissions [9]. This percentage is higher for high-income countries, ranging up to 10% of all emissions in the United States originating from healthcare [10]. If the global health sector were a country, it would be the fifth largest emitter of GHGs on the planet [9]. The limits to global resource consumption and waste production is are known as the planetary boundaries [11]. Currently, not a single country in the world meets the basic needs for its citizens in a sustainable way, i.e. remaining within these planetary boundaries [12].

The effects of climate change on human health are becoming more and more apparent and have already been labelled the biggest threat to global health in the 21st century [13–16]. This leads to the paradoxical situation where healthcare is both responding to the disease burden created by climate change, but is simultaneously aggravating the problem. This paradoxical role might explain the relatively low awareness regarding the health sector as a major emitter and polluter [17]. It urges us to rethink how we can transform healthcare into a sustainable industry.

The delivery of anaesthetic care is a sizable contributor to healthcare's footprint. The operating room generates 20–30% of hospital waste [18] and anaesthetic gases alone account for 2% of the total impact of the UK National Health Service [19]. This issue was previously introduced in this journal [20]. The current aims to update this publication and provide practical recommendations for anaesthesiologists that want to transform their own practice in an evidence-based way. 

**Box 1:**
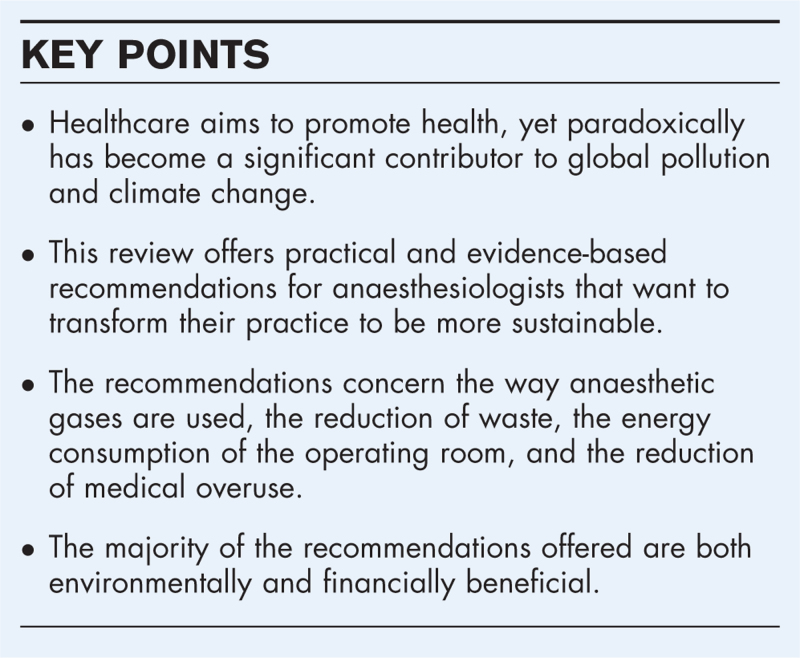
no caption available

## SUSTAINABILITY AND THE OPERATING ROOM

In the last decade, an increasing number of physician-initiated projects arose that aim to reduce healthcare's contribution to climate change, showing that many anaesthesiologists care about reducing their footprint [21,22^▪▪^]. The footprint of the operating room (OR) can be divided into three major scopes [23]. Scope 1 concerns direct emissions, mainly consisting of anaesthetic gases, scope 2 concerns indirect emissions from energy generation, and scope 3 includes all indirect emissions from up and downstream activities (i.e. supply chain and waste processing).

Reducing the environmental footprint from energy consumption can be achieved in three ways (‘the trias energetica’): minimising energy consumption, sustainable energy generation and efficient use of fossil fuels. Healthcare professionals on the operating room should primarily focus their attention on optimising heating, ventilation and air conditioning (HVAC) systems, because these are responsible for up to 90–99% of the energy demand [23]. General measures such as light-emitting diode (LED) lighting, green energy procurement, hospital thermal isolation, or photovoltaic panels are not specific to the operating theatre and considered out-of-scope for this paper.

The most frequently used method to compare footprints of products and processes is the life cycle assessment (LCA) [24]. These assessments are also called ‘cradle-to-grave analyses’ because they calculate the complete footprint of a medical device or drug, including the raw materials, manufacturing, packaging, distribution, usage and eventual waste processing. A comprehensive LCA database for medical devices and drugs has been proposed as a crucial tool in reducing healthcare's footprint [17], but is not yet available. Other important principles include the standardisation of sustainability metrics and the revision of infection control standards which often drive nonevidence-based uptake of single use disposable devices and HVAC systems. A combined approach can reduce the carbon footprint of an average laparoscopy by 80% [25]. Environmental sustainability should be an accepted part of decision-making at an organisational level, as well as part of an anaesthesiologist's education, research and quality improvement efforts [22^▪▪^].

## MEDICAL OVERUSE AND RESEARCH WASTE

Medical overuse is one of the major problems of modern medicine [26–28]. It simultaneously leads to too much care for healthy people, and not enough care for sick people [29]. It worsens health inequality and harms both people that need treatment, and those that do not. The idea that more care leads to better outcomes is deeply ingrained in the system [30]. However, cumulative care follows the ‘law of diminishing returns’ introduced above. Increasingly more care produces increasingly less patient benefit until it eventually causes more harm than good. In healthcare, including anaesthesiology and intensive care medicine, doing less is often just as good, or better, for patient outcomes. Examples from recent publications include lower haemoglobin targets as an infusion trigger [31], lower targets for oxygen saturation during mechanical ventilation [32] and lower intraoperative blood pressure [33]. It is estimated that only 32% of clinical practice is anaesthesiology is backed by level I or II evidence (i.e. at least one properly designed trial) [34].

Meanwhile, unrealistically positive effects of novel interventions are often assumed in anaesthesiology research. For example, some of the largest trials in anaesthesiology are based on bold assumption that interventions can lead to a 30% reduction in postoperative cancer recurrence [35] or a 53% reduction in postoperative myocardial injury [36]. Research waste, just like clinical waste, is detrimental to the environmental efforts and financial means of hospitals [37]. Being realistic about this is not nihilistic, but protective against potentially harmful care and the waste of resources. Hubris has been called the greatest foe of the modern clinician [38]. Eliminating medical overuse presents a largely untapped potential to reduce environmental harm, while also improving patient care and relieving some of the financial and personnel strain on healthcare systems [39^▪^].

## PRACTICAL RECOMMENDATIONS

The following section of this review proposes a series of practical recommendations to reduce the environmental footprint of the operating room. We have deliberately not tried to be exhaustive, but aimed to highlight seven evidence-based recommendations that are relatively easy to implement and have high potential impact. The financial consequences have also been reviewed.

*Recommendation 1: Use total intravenous anaesthesia over inhalation-based anaesthesia.*

For the first general anaesthesia administration, performed on October 16, 1846 by the dentist William Morton [40], the volatile substance diethyl ether was inhaled from a drenched cloth and anaesthesiologists have used volatile and gaseous substances to administer anaesthesia ever since. In the past years, this practice has come under increased scrutiny with respect to its environmental footprint. The current generation of halogenated anaesthetics all have the chemical properties of greenhouse gases with relatively high potency. Expressed as the 20-year Global Warming Potential (GWP_20_), 1 kg of sevoflurane equals the emission of 440 kg CO_2_ and one kg of desflurane equals 6810 kg CO_2_[41]. Most of these substances are hardly metabolised and enter the atmosphere after use where they have lifetimes of 1.1 to 14 years. One study measured atmospheric presence of anaesthetics in Northern Pacific and the Swiss Alps and on Antarctica and found increasing concentrations in these very remote areas [42]. As an alternative to volatile anaesthetics, total intravenous anaesthesia (TIVA) may be employed. The GHG impact of, for example, propofol is very small and stems from the energy needed to operate the syringe pump [43]. A life cycle analysis showed that the carbon footprint of anaesthesia for a hysterectomy varied greatly between 0.001 kg CO_2_-eq(uivalent) for propofol versus 505 kg CO_2_-eq for desflurane. Although carbon footprint is greatly affected, patient outcome did not differ between the groups. The fact that choice of anaesthetic may only have minimal influence on patient outcome has been underlined in recent years where large randomised controlled trials have not demonstrated any significant impact on mortality after cardiac surgery [44] or cancer recurrence [35]. Also, ‘soft-outcome’ including postoperative cognitive dysfunction, postoperative nausea and vomiting and recovery time does not differ between TIVA and inhalational anaesthesia in vulnerable groups [45,46]. With regard to financial cost, one study compared propofol-based to inhalation-based anaesthesia for noncardiac surgery in the United States and concluded overall costs were 11–12% lower with propofol, mainly because of a reduced incidence in postoperative nausea and vomiting and earlier discharge from recovery [47].

*Recommendation 2: Use ultra-low fresh gas flow (<0.5 l min* ^*−1*^ *) during inhalation based anaesthesia and higher flows (4–6 l min* ^*−1*^ *) during total intravenous anaesthesia.*

Since emission of volatile anaesthetics is directly related to fresh-gas flows (FGF) employed, it is self-evident to reduce FGF as low as possible. When reducing FGF from 2.0 l min^−1^ to 0.5 l min^−1^ one study found that sevoflurane consumption could be decreased by 60% [48]. In countries where regulations do not allow these ultra-low FGF during sevoflurane-based anaesthesia, an alternative CO_2_-absorber may be used to achieve this. Low FGF can also be adopted during mask-induction as one study showed that sevoflurane consumption can be halved by employing a specific low-flow protocol without affecting induction time or reaction to airway manipulation in children [49]. With regard to TIVA, optimal FGF is related to the cost and footprint of CO_2_-absorber exhaustion on the one hand, versus the energy requirements to produce medical gases such as oxygen on the other. An LCA from Australia suggests that cost can be reduced with 93% when switching from 1 to 6 l min^−1^ with minimal effect on carbon footprint [50]. However, as others have argued [51], energy generation in Australia is predominantly coal-based and among the most carbon intensive in the world. In regions with more favourable energy mixes, higher FGF generates not only financial, but also environmental benefit.

*Recommendation 3: Anaesthetic gas capturing technology is still largely unproven and may have limited environmental benefit, while it increases cost.*

To prevent emission of volatile anaesthetics to the environment, different technologies have been developed and tested. Most commercial devices contain proprietary elements that have not been published in scientific literature. Generally, the chemical principle behind these devices is adsorption to activated carbon, zeolite, molecular sieves, or silica gel. An in-vitro study compared all these different substances and found acceptable maximal adsorption efficiency for sevoflurane (89%) and isoflurane (93%), but limited efficiency for the most important greenhouse gas, desflurane (77%) [52]. Adsorption is a reversible process and offers the potential to recapture the substance for reuse. The only technology that was examined in-vivo in a peer-reviews study measured adsorption efficiency in 80 consecutive desflurane-based general anaesthetics. From the total 6902 g administered desflurane, only 2509 g was absorbed into the active charcoal (CONTRAFluran, ZEOCYS, Luckenwalde, Germany) and ultimately 1727 g could be recaptured for reuse, yielding a total recapturing percentage of 25% [53^▪▪^]. This is not efficacious enough to justify investing in this technology, especially because the environmental footprint of the entire process is still unknown and the reuse of recaptured medication is not (yet) allowed under current regulation.

*Recommendation 4: In the clinical setting, epidural or intravenous analgesia during labour is preferred over nitrous oxide inhalation.*

Various options exist for labour analgesia. Most common methods are epidural analgesia, inhaled nitrous oxide (N_2_O), and the intramuscular of intravenous injection of opioids (e.g. patient controlled analgesia (PCA) with remifentanil). Availability of these options varies greatly between countries and hospitals. In the UK, it is recommended that N_2_O should be available in all birth settings, which has led to 30% of all GHG emissions of anaesthetic services attributable to N_2_O administration in the labour setting [54^▪^]. A recent study compared the total carbon footprint in CO_2_-eq of 4-h periods of different modes of labour analgesia. The results showed that N_2_O administration results in 237 kg CO_2_-eq compared with 1.2 kg CO_2_-eq for epidural bupivacaine and 0.75 kg CO_2_-eq for remifentanil PCA [54^▪^]. Seeing that both epidural and remifentanil provide superior analgesia over N_2_O [55], at a fraction of the footprint, these should be the preferred options for the management of labour pain in the clinical setting.

The financial comparison is dependent upon the fee of getting an anaesthesiologist involved to administer the epidural. This may be variable across different countries, but the epidural is likely the more expensive option compared with intravenous opioids and nitrous oxide.

*Recommendation 5: Properly designed air treatment systems save energy and reduce cost.*

Installation and operation of HVAC systems in ORs is costly and produces a sizable environmental footprint due to their high energy demand. Proper design, as well as retrofitting existing systems with a focus on energy conservation can reduce energy consumption by 65%. Effective measures include recirculation, thermal wheels, glycol coil heat recovery and heat recovery chiller [56]. Which measures are most beneficial depends on geolocation of the hospital and the local situation. Furthermore, temperature settings closer to outside temperatures reduce cost and energy consumption.

With respect to HVAC design, laminar airflow displacement ventilation (LAF) systems are able to increase air quality over conventional mixed ventilation systems at the expense of much greater energy consumption and higher installation cost [57,58^▪^,^59^,^60^]. However, evidence that these systems reduce the incidence of surgical site infections (SSIs) is lacking. Although the benefit for LAF systems in joint replacement surgery is still under debate, recently published guidelines ceased to recommend the use of these systems for general use in ORs [61].

*Recommendation 6: Unoccupied set-back of air treatment systems can save up to 70% of energy.*

When not in use, HVAC systems in ORs can be turned down without affecting patient safety. In the United States, one study showed that reducing air exchanges on 19 of their 22 operating theatres, lowered energy demand with 50% [23]. A study in Taiwan found energy savings of 70% after unoccupied set-backs were implemented [62]. This measure is easy to employ and requires no structural changes or upfront investment (only minimally when occupancy sensors are used).

*Recommendation 7: Reusable equipment is almost always associated with a lower environmental impact and lower costs compared with single-use disposables.*

For most materials used in the OR, both a reusable and a single-use disposable option exist. Being able to use equipment multiple times has the obvious benefit of creating less waste and requiring less raw materials, but it has long been unclear whether the environmental burden of the disinfection procedures of reusable equipment offsets these benefits. We aimed to review all available publications that compared the impact of reusable and disposable equipment used in the OR. We found 15 studies which are listed in Table 1. It unanimously shows that reusable equipment produces a lower environmental impact. The only deviating results came from the three studies performed in Australia [63–65]. As discussed in recommendation 2, electricity generation is almost exclusively coal-based in Australia. Since the sterilisation process is electricity-intensive, this negates the environmental gains. Two of the three studies have recalculated their results for a European or an American power mix [64,65]. This resulted in a clear environmental benefit for the reusable option. A similar analysis is not available for the third publication [63], but a comparable benefit of reusable equipment is to be expected.

**Table 1:** Various life cycle assessment studies investigating disposable versus reusable alternatives of frequently used anaesthetic and surgical equipment. Environmental burden is generally lower for reusable equipment, although in countries with coal-based electricity generation, such as Australia, environmental impact of disinfection and sterilization can offset these environmental gains

First author	Publication year	Subject	Lowest environmental impact	Lowest cost
Adler [66]	2004	Laparoscopy instruments	Reusable	Reusable −94%
Davis [63]	2018	Ureteroscope	Australia: similar impact	N/A
Donahue [67]	2020	Vaginal specula	Reusable	N/A
Eckelman [68]	2012	Laryngeal mask airway	Reusable	Reusable −17%
Friedericy [69]	2022	Sterilization canister	Reusable	N/A
Grimmond [70]	2020	Needle container	Reusable	Reusable −19%
Ibbotson [71]	2013	Scissors	Reusable	Reusable −45%
Leiden [72]	2020	Spinal fusion set	Disposable	N/A
McGain [73]	2010	Anaesthetic tray	Reusable	Reusable −51%
McGain [63]	2012	Central venous catheterisation kits	Australia: disposable; USA/Europe: reusable	Reusable -27%
McGain [64]	2017	Anaesthesia equipment (face mask,	Australia: disposable; USA/Europe: reusable	Reusable −46%
Rodriguez [74]	2022	Vaginal specula	Reusable	N/A
Sanchez [75]	2020	Blood Pressure Cuffs	Reusable	Reusable −89%
Sherman [76]	2018	Laryngoscope	Reusable	Reusable blades −59%; handles −78%
Vozzola [77]	2018	Surgical drapes and gowns	Reusable	N/A

N/A, not available.

Of the 15 publications, nine included a financial analysis. For all nine comparisons, the reusable option came with a cost reduction of between 17% and 94%. Additionally, there is no indication that reusables carry an increased risk of infection [58^▪^].

## CONCLUSION

The excessive growth of the healthcare sector has created an industry that is responsible for a significant part of global environmental pollution. This is at odds with healthcare's aim of improving health, urging us to transform healthcare into a sustainable industry. In this review, multiple recommendations are presented that can help anaesthesiologists to improve the environmental impact of their practice in the OR. Because environment and economy are inextricably entwined we have tried to delineate the financial impact of each recommendation. In most cases, the more sustainable choice resulted in cost savings as well.

## Acknowledgements

*None.*

### Financial support and sponsorship

*None.*

### Conflicts of interest

*There are no conflicts of interest.*

## REFERENCES AND RECOMMENDED READING

Papers of particular interest, published within the annual period of review, have been highlighted as:

▪ of special interest
▪▪ of outstanding interest
